# Allergen sensitization linked to climate and age, not to intermittent-persistent rhinitis in a cross-sectional cohort study in the (sub)tropics

**DOI:** 10.1186/2045-7022-4-20

**Published:** 2014-06-04

**Authors:** Désirée Larenas-Linnemann, Alexandra Michels, Hanna Dinger, Kijawasch Shah-Hosseini, Ralph Mösges, Alfredo Arias-Cruz, Marichuy Ambriz-Moreno, Martín Bedolla Barajas, Ruth Cerino Javier, María de la Luz Cid del Prado, Manuel Alejandro Cruz Moreno, Roberto García Almaráz, Cecilia Y García-Cobas, Daniel A Garcia Imperial, Rosa Garcia Muñoz, Dante Hernández-Colín, Francisco J Linares-Zapien, Jorge A Luna-Pech, Juan J Matta-Campos, Norma Martinez Jiménez, Miguel A Medina-Ávalos, Alejandra Medina Hernández, Alberto Monteverde Maldonado, Doris N López, Luis J Pizano Nazara, Emmanuel Ramirez Sanchez, José D Ramos-López, Noel Rodríguez-Pérez, Pablo G Rodríguez-Ortiz

**Affiliations:** 1 Hospital Médica Sur, Torre 2, cons.602, Puente de Piedra 150, Colonia Toriello Guerra; Delegación Tlalpan, México, DF 14050, Mexico; 2 Institute of Medical Statistics, Informatics and Epidemiology (IMSIE), University of Cologne, Cologne, Germany; 3 Mexican Study Group on Allergic Rhinitis and Skin Sensitivity. Hospital Médica Sur, Torre 2, cons.602, Puente de Piedra 150, Colonia Toriello Guerra; Delegación Tlalpan, México, DF 14050, Mexico; 4 University of Cologne, Cologne, Germany; 5 Centro Regional de Alergia e Inmunología Clínica, Hospital Universitario "Dr. José Eleuterio González" de la Universidad Autónoma de Nuevo León, Monterrey, Nuevo León; 6 Servicio de Alergia e Inmunología Clínica, División de Medicina Interna, Hospital Civil de Guadalajara "Dr. Juan I. Menchaca" Universidad de Guadalajara, Guadalajara, Jalisco, Mexico; 7 CEINTAP (centro de investigación y tratamiento del asma pediátrico), Hospital Regional de Alta Especialidad del Niño. Dr. Rodolfo Nieto Padrón, Villahermosa, Tabasco, Mexico; 8 Clinica Santa Cruz, Villahermosa, Tabasco; 9 Allergy Department, Hospital Infantil de Tamaulipas, Cd.Victoria, Tamaulipas, Mexico; 10 Inmunología y Alergia, UMAE Hospital de Especialidades, CMNO, IMSS, Guadalajara, Jalisco, Mexico; 11 Allergy Department, Hospital Médica Tec 100, Querétaro, Querétaro, Mexico; 12 Centro de Diagnostico y Tratamiento de Enfermedades Alérgicas y Asma de Toluca, Toluca, Estado de México; 13 Allergy and Clinical Immunology Department, Hospital of specializations CMN ‘Siglo XXI’, Mexico, DF, Mexico; 14 Coordination of evidence based medicine (EBM), Facultad de medicina. Universidad Autonoma de Queretaro, Querétaro, Querétaro, Mexico; 15 Clinical immunology & allergy department, Clínica Hospital San Jose, Cd.Obregón, Sonora, Mexico; 16 Allergy and immunology department, General Hospital of Cancun "Dr. Jesus Kumate Rodriguez', Cancun, Quintana Roo, Mexico; 17 Servicio de Alergia e Inmunología Clínic, Seguro Social, Hospital General de Zona No2, Potosí, Potosí, Mexico; 18 Pediatrics and Immunology, Autonomous university of Tamaulipas, H.Matamoros, Tamaulipas, Mexico; 19 Hospital Star Médica Mérida, Mérida, Yucatan, Mexico

**Keywords:** House dust mite, Pollen, Skin prick test, Allergic sensitization, Allergic rhinitis, Intermittent rhinitis, Persistent rhinitis, Seasonal, Perennial

## Abstract

**Background:**

Allergen exposure leads to allergen sensitization in susceptible individuals and this might influence allergic rhinitis (AR) phenotype expression. We investigated whether sensitization patterns vary in a country with subtropical and tropical regions and if sensitization patterns relate to AR phenotypes or age.

**Methods:**

In a national, cross-sectional study AR patients (2-70 y) seen by allergists underwent blinded skin prick testing with a panel of 18 allergens and completed a validated questionnaire on AR phenotypes.

**Results:**

628 patients were recruited. The major sensitizing allergen was house dust mite (HDM) (56%), followed by Bermuda grass (26%), ash (24%), oak (23%) and mesquite (21%) pollen, cat (22%) and cockroach (21%). Patients living in the tropical region were almost exclusively sensitized to HDM (87%). In the central agricultural zones sensitization is primarily to grass and tree pollen. Nationwide, most study subjects had perennial (82.2%), intermittent (56.5%) and moderate-severe (84.7%) AR. Sensitization was not related to the intermittent-persistent AR classification or to AR severity; seasonal AR was associated with tree (p < 0.05) and grass pollen sensitization (p < 0.01). HDM sensitization was more frequent in children (0-11 y) and adolescents (12-17 y) (subtropical region: p < 0.0005; tropical region p < 0.05), but pollen sensitization becomes more important in the adult patients visiting allergists (Adults vs children + adolescents for tree pollen: p < 0.0001, weeds: p < 0.0005).

**Conclusions:**

In a country with (sub)tropical climate zones SPT sensitization patterns varied according to climatological zones; they were different from those found in Europe, HDM sensitization far outweighing pollen allergies and Bermuda grass and Ash pollen being the main grass and tree allergens, respectively. Pollen sensitization was related to SAR, but no relation between sensitization and intermittent-persistent AR or AR severity could be detected. Sensitization patterns vary with age (child HDM, adult pollen). Clinical implications of our findings are dual: only a few allergens –some region specific- cover the majority of sensitizations in (sub)tropical climate zones. This is of major importance for allergen manufacturers and immunotherapy planning. Secondly, patient selection in clinical trials should be based on the intermittent-persistent and severity classifications, rather than on the seasonal-perennial AR subtypes, especially when conducted in (sub)tropical countries.

## Background

Allergic rhinitis has been of increasing importance over recent decades, because of its rise in prevalence [1] and linked co-morbidities, including asthma and chronic upper respiratory tract infections [2]. Detection of the sensitizing allergen allows for a more complete therapy, as allergen avoidance should form an integral part of the treatment. Moreover, recognition of the sensitizing allergen is essential to the adequate preparation of specific allergen immunotherapy, an approach directed at the cause of the disease [23,5].

This manuscript explores allergen sensitization patterns in patients with AR in a country in which several climate zones can be differentiated, varying from sub-tropic to tropic, and thus leading to a sensitization pattern different from that found in Europe and the United States (US).

Mexico is situated between latitude 14.32° and 32.46° north of the Equator and as such falls partly in the subtropics and partly in the tropics. A striking humidity-gradient can be detected from the hot, dry North through the semi-dry temperate center of the country, to the humid south-eastern tropical part. These characteristics together with the varying altitude divide the country according to the National Geographic Institute (*Instituto Nacional de Estadística y Geografía*, INEGI) in six climate zones [6]. The ISAAC studies found a prevalence of rhinitis in Mexican children between 11.6-15.4% and asthma-like symptoms between 8.5-15.6% [1,7,8]. Allergic rhinitis risk factors and economic impact have been documented in that country [9,10]. In the present article we describe how allergen sensitization is distributed over the whole country and how it is linked to allergic rhinitis phenotypes, age and climate zones. Detailed AR phenotype data will be presented in a separate manuscript [11].

## Methods

This study was carried out as a prospective, cross-sectional, nationwide, multicenter study.

### Climate zones and patient selection

**Figure 1:**
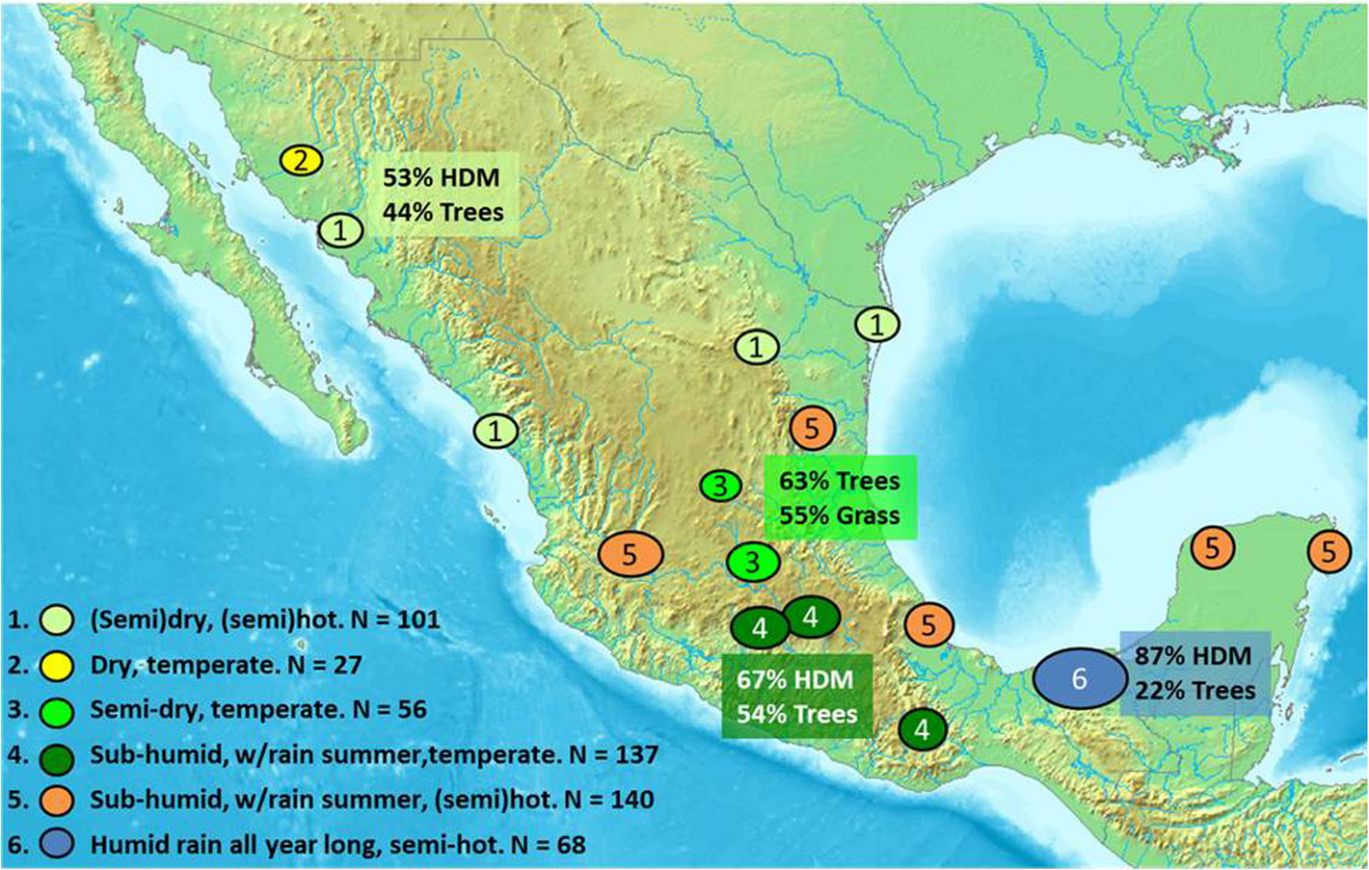
**Center distribution and allergen sensitivity throughout the Mexican republic.** Location of the centers from which allergic rhinitis subjects were recruited, and their distribution over the six different climate zones in Mexico. The area from which subjects from the tropical zone were recruited is broad, reflected by the large dot.

Twenty-six study centers were selected, distributed over the six climate zones across Mexico. The division of the climate zones was according to the official INEGI assignation (http://www.inegi.org.mx/geo/contenidos/recnat/clima/default.aspx and related charts), see Figure 1. Consecutive patients (2-70 years) with rhinitis symptoms were pre-selected if they had symptoms compatible with allergic rhinitis, e.g. symptoms exacerbating during a specific season and/or on exposure to certain allergen(s). After testing positively with the routine SPT of the center, subjects were asked to participate in the study. Thus, recruited patients were those routinely diagnosed as having allergic rhinitis by the allergists. The patients (and for the pediatric patients the parents/legal guardian) gave written informed consent. The study was evaluated and approved by an independent Ethics Committee. Subjects were excluded if they had any contra-indication for undergoing skin prick testing or if they had had immunotherapy within the previous five years. The elimination criteria were an invalid study SPT or if the criteria for the study diagnosis of rhinitis –see below- were not met.

### Allergic rhinitis symptom phenotypes

Eligible subjects were invited to fill out a validated questionnaire on rhino-conjunctivitis symptoms [12], which allows classification of allergic rhinitis (AR) as seasonal-perennial (SAR-PAR) on one hand and according to the ARIA classification as intermittent (IAR) versus persistent (PER) and mild versus moderate-severe on the other hand [13]. For children below 12 years, the carer completed the questionnaire. A subject was defined as suffering from rhinitis if at least two of the four enquired rhinitis symptoms (congestion, rhinorrhea, pruritus, sneezing) were positive and conjunctivitis if one of three conjunctivitis symptoms (erythema, pruritus, lacrimation) were present. AR was classified according to the questionnaire data as completed by the patients and following the ARIA guidelines as intermittent (symptoms appearing <4 days per week or <4 wks.) or persistent.

### Skin prick testing

Once the presence of rhinitis/rhinoconjunctivitis was confirmed, subjects underwent the study skin prick test with a blinded set of sixteen of the most common [14] allergens in Mexico (*Dermatophagoides* mix^stand^*,* cat^stand^, *Poa pratensis*^stand^, *Phleum pratense*^stand^, *Cynodon dactylon*^stand^, *Ambrosia*^stand^, profilin, peach: ALK-Abelló, Madrid, Spain,^stand^ = standardized; *Blomia tropicalis*^stand^: Biocen, La Havana, Cuba; Blatella germanica/Periplaneta americana mix, *Fraxinus excelsior*, *Quercus ilex*, *Prosopis*, *Cupressus sempervirens*, *Chenopodium album*, *Heliantus*, *Alternaria alternata*, *Aspergillus fumigatus*: Allerquim, Mexicocity, Mexico). *Dermatophagoides* mix is a 50-50% mix of *Dermatophagoides pteronyssinus and farinae.* The English names of the allergens are: house dust mite, cat, Kentucky bluegrass, Timothy, Bermuda grass, Ragweed, Blomia, cockroach mix, Ash, oak, mesquite, cypress, lamb’s quarter, sunflower, Alternaria, Aspergillus. Blinding of SPT extracts was carried out at a central site, not involved in patient recruitment. Technicians applying the SPT, physicians and patients were all blinded to the content of the vials, as was the statistical team. The skin testing was done in duplicate. With an ALK-lancet (ALK-Abelló, Madrid, Spain), the skin was penetrated, passing through each drop of allergen extract at 90°. Personnel that applied the test in the centers passed a previously described proficiency test [15] (coefficient of variation <20%) before recruiting the first patient. The results were read at 15 min by drawing around the perimeter of the wheal with a ballpoint pen, placing transparent tape against the skin and transferring the prints to the data collection sheet. A test was considered positive if the wheal surface ≥ 5 mm [2] and if it met the criteria of a valid test (negative control <5 mm^2^, positive control ≥5 mm^2^) [16].

### Data analysis

Using all the above data, we analyzed SPT positivity allergen-clusters (‘grasses’, ‘trees’, etc.) and individual allergens nationally. With house dust mite (HDM) we refer to only *Dermatophagoides pteronyssinus and farina*e, not Blomia. Likewise, the frequency distribution of skin test sensitivity to certain allergen-clusters and its relation to the following factors was examined: rhinitis symptom phenotypes, age and climate zones.

### Statistical analysis

The programmed sample was 90 patients per climate zone. Sample size calculation [17] was based on an estimated population size of 500,000 AR patients (500 practicing allergists seeing approximately 1,000 allergic rhinitis patients/year), an estimated frequency of SPT positivity for HDM of 55% with a confidence level or 95% and a confidence interval of 10% (standard error 0.05). For the allergens with an estimated frequency of SPT positivity close to 25% (or 75%), 73 patients per climate zone would have been sufficient to achieve the identical confidence interval. Pearson’s χ squared tests were used to compare the frequency of the SPT positivity between the climate zones, the age-groups and the phenotypes of rhinitis (if necessary Yates’ correction was applied). Differences in wheal size were compared with the Mann-Whitney rank test.

## Results

Six hundred twenty-eight patients were recruited. 91/628 patients were excluded because of a too large negative control (83) or other SPT errors. Nationwide a total of 529 patients with skin prick test positivity and rhinitis and/or conjunctivitis symptoms were included (aged 2-68 years, mean age 21.8 years, 48.2% male). Their distribution over the six climate zones can be seen in Figure 1. In the tropical zone 6 there were more subjects in the 2-11 year age-group, see Table 1.

**Table 1:** Distribution of subjects over the tropical and subtropical zones

	**Subtropics (Zones 1-5)**	**Tropics (Zone 6)**
**Total**	**457**	**68**
Gender		
Male	216 (48%)	36 (53%)
Female	239 (52%)	32 (47%)
Age		
2 to 11 years	111 (24%)	46 (68%)
12 to 17 years	78 (17%)	13 (19%)
18+ years	268 (58%)	9 (13%)

### Frequency of skin prick test positivity Nationwide

**Figure 2:**
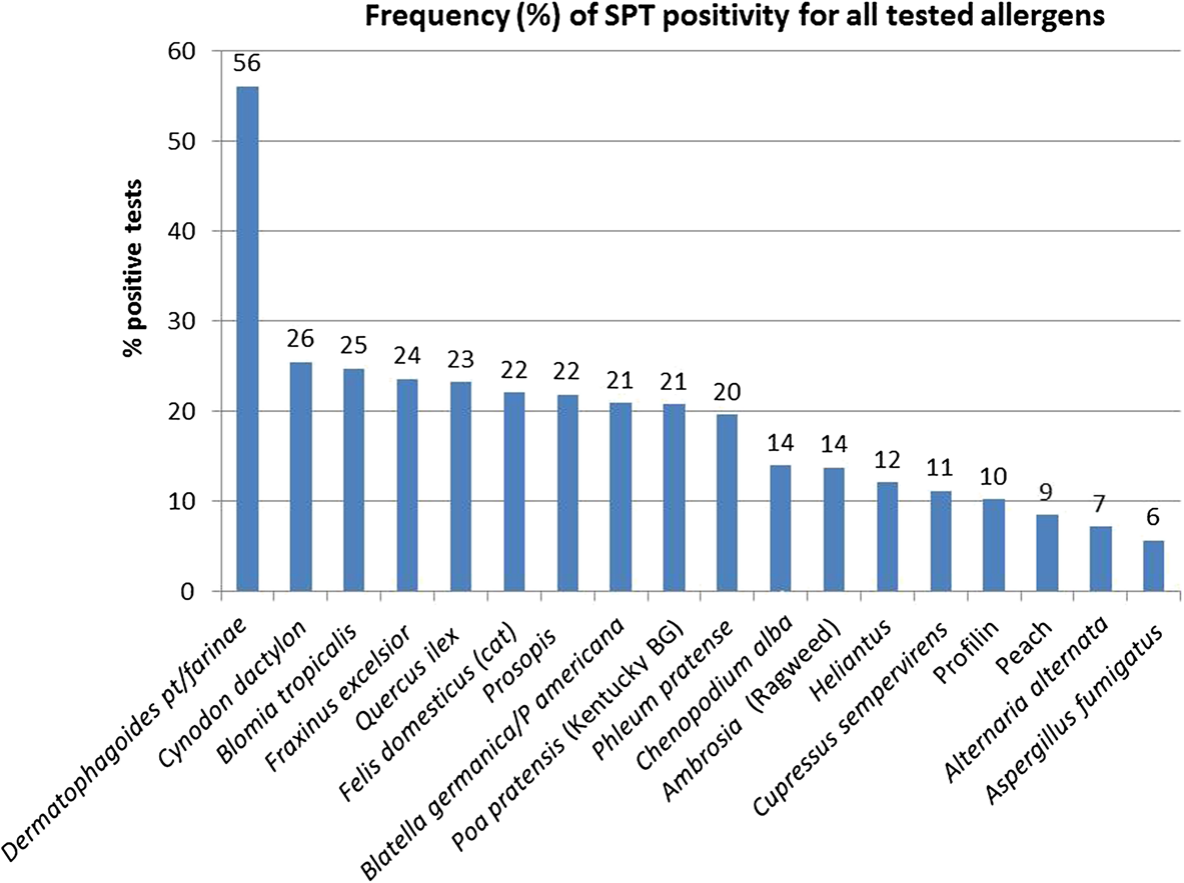
**Nationwide skin prick test (SPT) positivity for all tested allergens.** Percentage of skin prick test positivity per allergen of all included subjects, nationwide

Nationally, SPT positivity was mostly found for mites (proportion 0.599, 95% confidence interval (CI) 0.55-0.64), followed by tree pollens (0.442, 95% CI: 0.40-0.48), grass pollens (0.338, 95% CI: 0.30-0.38), weed pollens (0.265, 95% CI: 0.23-0.30) and finally molds (0.113, 95% CI: 0.09-0.14). As such the frequency of SPT positivity for individual allergens could be divided into three groups (Figure 2). Firstly, the most frequent allergen, *Dermatophagoides* mix (*pteronyssinus* + *farinae*), with a positive SPT in more than 56% of the patients. Secondly, the ‘frequent’ allergens, with percentages of SPT positivity between 20-26% -cat allergen is in this group- and thirdly, the less frequent ones (SPT positivity from 6 to 14%). As for the mean wheal size in SPT positive patients, there was large variation. The largest wheals were produced by *Cynodon dactylon*, followed by profilin, peach and *Aspergillus. Dermatophagoides* mix produced a mean wheal size in the middle range (Additional file 1: Figure S1). As such, the most frequent allergen was not the one producing the largest wheals.

### Frequency of skin prick test positivity and wheal sizes: age groups

**Figure 3:**
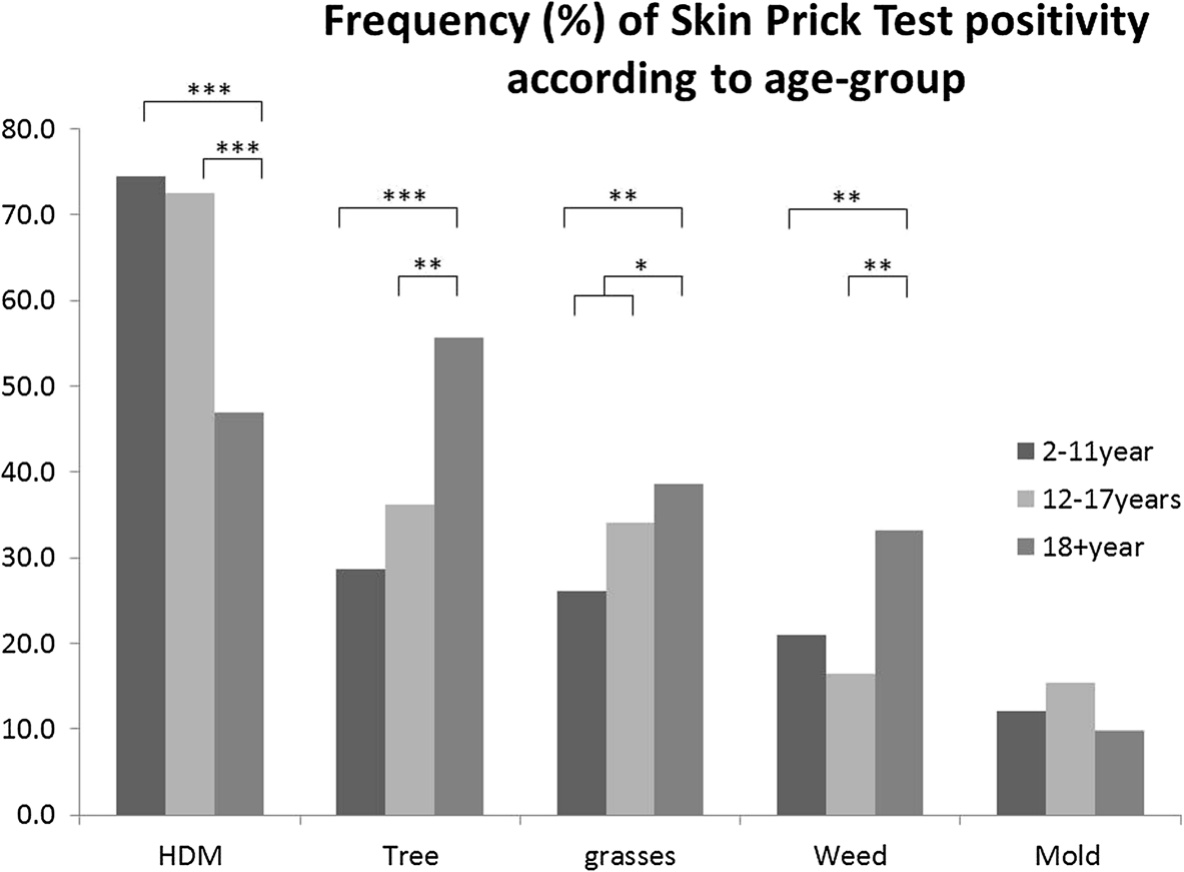
**Skin prick test positivity according to age-groups.** Frequency distribution of skin prick test positivity per allergen group, according to age-groups: children (2-11y), adolescents (12-17y) and adults (18y and up). * = p < 0.05, ** = p < 0.01, *** = p < 0.005.

Figure 3 shows the skin prick test positivity according to the age-groups. Adults (age 18+ years) had more SPT positivity for pollens of trees (p < 0.0001) and weeds (p < 0.0005), as compared to the younger age groups. However, SPT positivity for mites was statistical significantly more frequent in the 2-11 and 12-17 year age-groups, as compared to the adult subjects. These differences were maintained even if only subjects from the subtropic zones 1-5 were taken into account (HDM sensitization 2-11 years: 68%; 12-17 years 71%; 18 + years 46%; p < 0.0005 for comparisons of children-adults and adolescents-adults). For subjects from the tropical zone 6 the same trend could be seen, but because of small numbers the differences did not reach statistical significance (HDM sensitization 91%, 85% and 67%, respectively for children-adolescents-adults). We also compared the mean wheal size of SPT positive subjects between age-groups (see Additional file 2: Table S1 and S2). Mean wheal size of the 2-11 year age-group were generally smaller than the other two age-groups, reaching statistical significance for several allergens. In general there was no difference between the 12-17 year and 18+ year subjects, with even larger wheals in the adolescents as compared to the adult subjects for both mites (*Dermatophagoides*: p = 0.023; blomia: p = 0.026).

### Frequency of skin prick test positivity and wheal sizes: allergic rhinitis phenotypes

**Figure 4:**
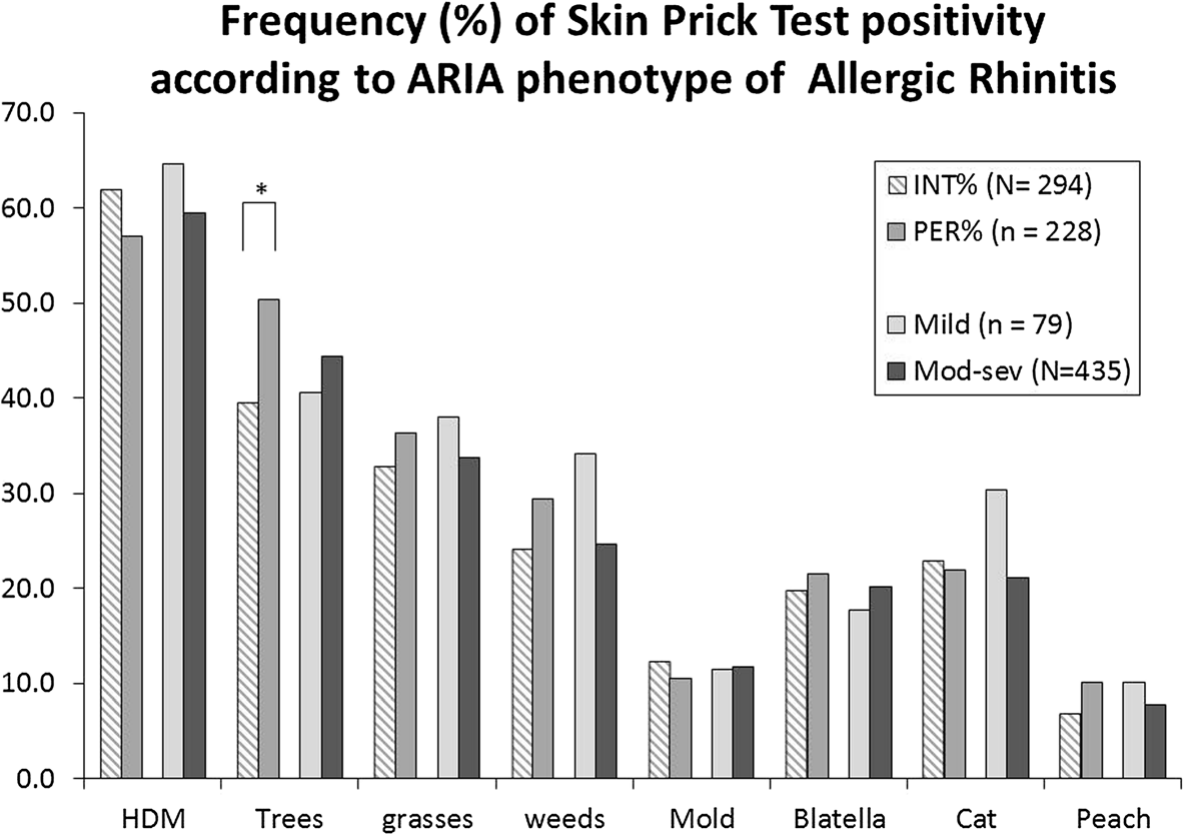
**Skin prick test positivity according to allergic rhinitis phenotypes (intermittent-persistent, mild-moderate/severe).** Frequency distribution of skin prick test positivity per allergen group, according to ARIA allergic rhinitis phenotypes: intermittent or persistent and mild or moderate-severe. * = p < 0.05.

**Figure 5:**
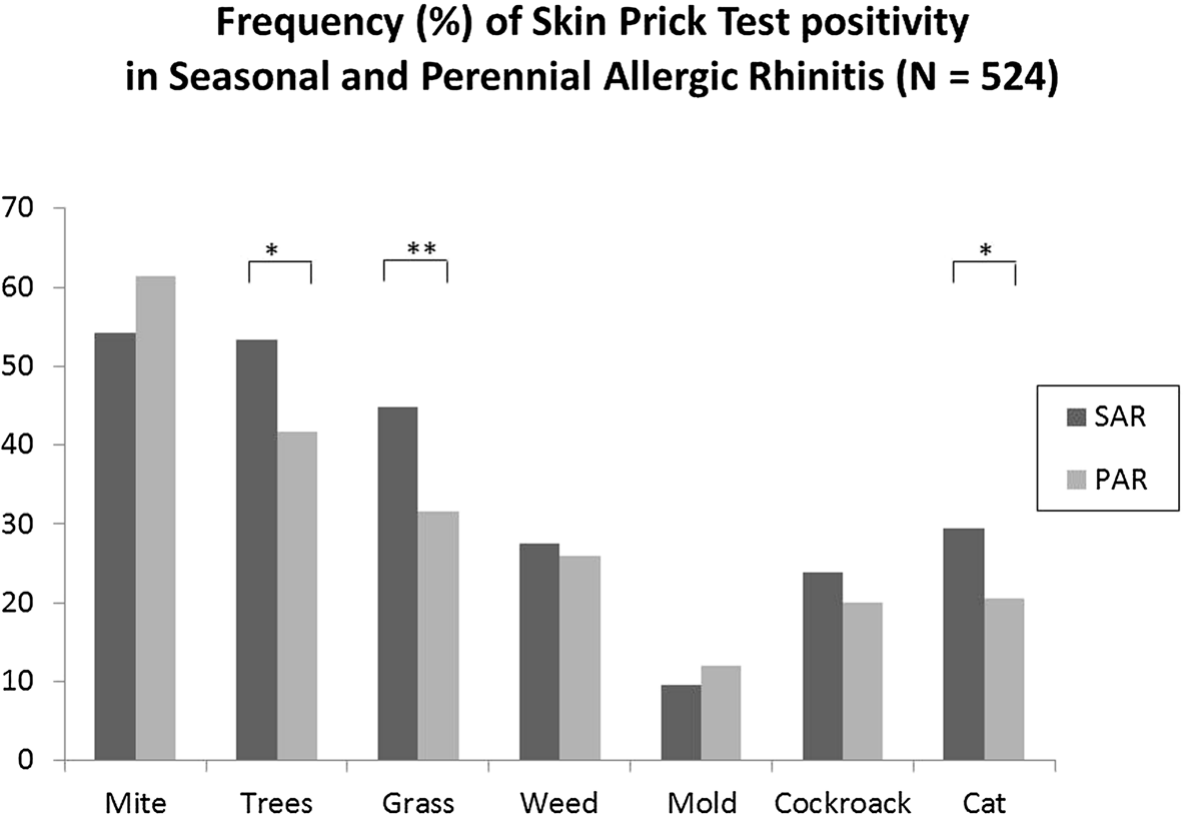
**Skin prick test positivity according to seasonal-perennial allergic rhinitis.** Frequency distribution of skin prick test positivity per allergen group, according to allergic rhinitis phenotypes as per the previous classification: seasonal or perennial. * = p < 0.05, ** = p < 0.01.

There was no difference between intermittent (IAR) and persistent (PER) allergic rhinitis in the frequency of SPT positivity for any allergen tested, except for trees. Patients with PER had more frequent SPT positivity to trees as compared to patients with IAR (p = 0.012, Figure 4). For mild versus moderate-severe AR no difference could be found for any allergen. Comparing SAR with PAR patients, grass, tree and cat SPT positivity was more frequent in SAR patients (Figure 5).

As for wheal size comparing IAR versus PER and mild versus moderate-severe patients, no differences could be found, with one exception: PER patients presented a larger wheal surface for *Alternaria*. PAR patients had larger wheals than SAR patients for *Prosopis*, *Chenopodium*, cockroach mix and profilin.

### Frequency of skin prick test positivity per climate zone

**Figure 6:**
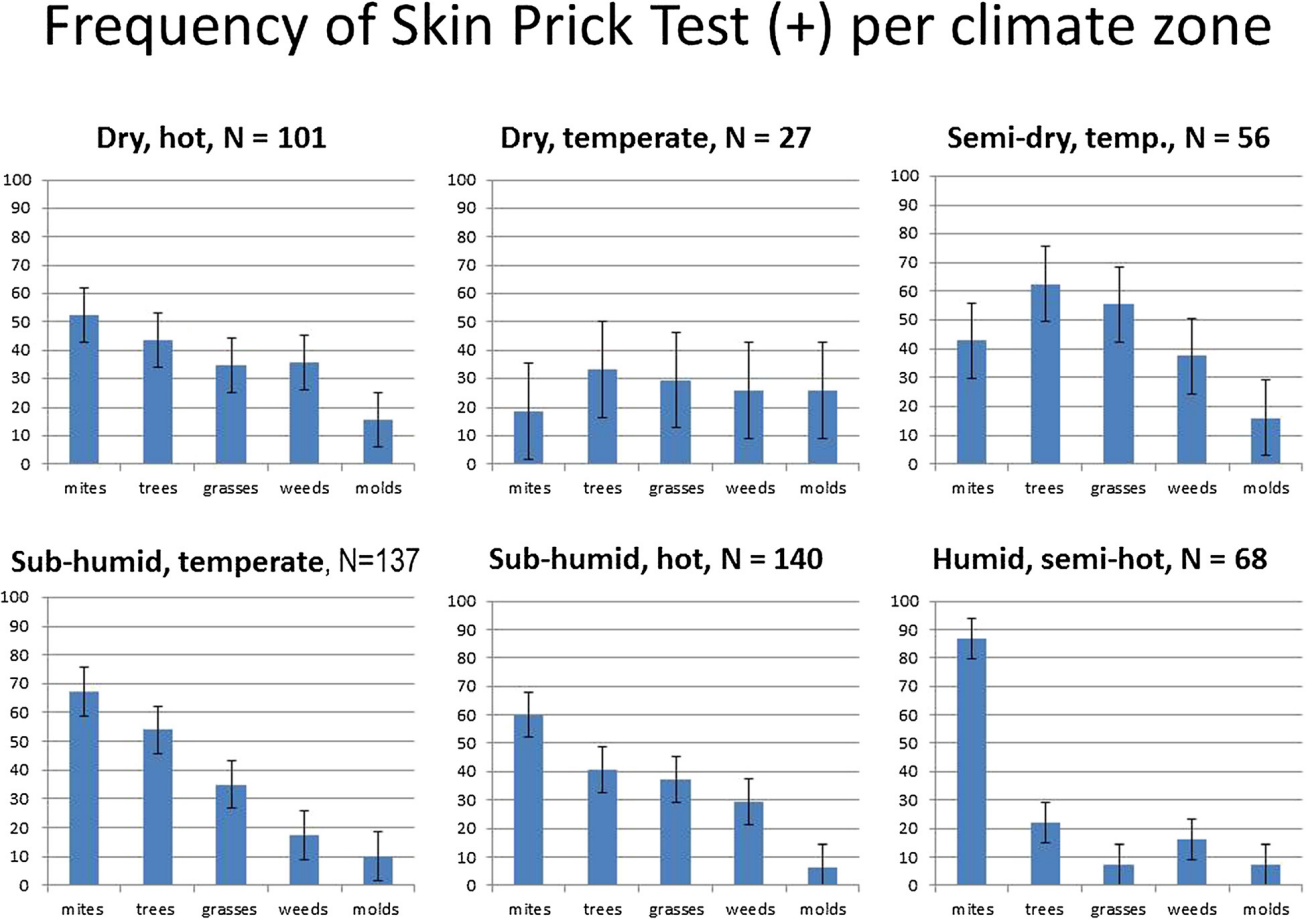
**Skin prick test positivity according to climate zones.** Percentage of skin prick test positivity per allergen cluster according to the six climate zones.

Statistically significant differences of positive SPT in a specific zone as compared to the rest of the country are documented in Figure 6 and Table 2 compares sensitization between the climate zones. In the dry-hot and the (sub)humid zones HDM was the most frequent allergen, followed by trees. In the dry North of Mexico sensitization to molds and weeds (and consequently also to pan-allergens such as peach –rich in lipid transfer protein- and profilin) are high. In the tropical zone 6, warm with rainfall all year long, almost all patients are sensitized to HDM, leaving only 20% of the patients with some additional pollen allergy. However, for the patients in the middle of the country, living in the agricultural zone with a (semi)-dry temperate climate, both tree and grass pollen sensitization is more common than HDM sensitization. Cockroach sensitization is important all over the country.

**Table 2:** Statistically significant differences* linked to Figure6

	**(Semi)dry, (semi)hot**	**Dry, temperate**	**Semi-dry, temperate**	**Subhumid, temperate**	**Subhumid, (semi)hot**	**Humid, semihot**
Mites		< 0.0001	<0.006	**<0.05**		**< 0.0001**
Grasses			**0.0003**			< 0.0001
Trees			**<0.004**	**0.007**		< 0.0001
Weeds	**0.02**		**<0.05**	(-0.058)		<0.04
Molds		**0.01**			0.03	
Cockroach						
Cat			**0.01**			0.002
Peach		**< 0.0001**		0.01*		0.05*
Profilin		**< 0.0001**		*Yates’ correction	0.006*	

*Pearson X-square tests were used, if necessary with Yates’ correction.

Proportion of skin prick test positivity (SPT (+)) in each climate zone as opposed to the rest of the country Normal font = % of SPT (+) is lower than country mean, bold font = % of SPT (+) is higher than country mean.

## Discussion

This article’s main accomplishments are four. Firstly, it delivers a detailed description of allergen sensitization patterns in a (sub)tropical country, different from those found in Europe and the US. Secondly, it demonstrates that in this setting allergic sensitization is not linked to a specific AR symptom phenotype, with the exception of SAR in which patients had slightly more tree and grass pollen positivity. Thirdly, it reveals that in this study population children have a higher rate of house dust mite sensitization and that pollen sensitization is more frequent in adult patients. Finally, it shows allergen sensitization profiles can vary within one country. Thus, although allergen sensitization did not vary with AR phenotypes, it is linked to climate zones, with grass and tree pollen being the most common sensitizing aeroallergen in the sub-tropical, temperate mid-country agricultural region, as opposed to *Dermatophagoides* being by far the most important sensitizing allergen in the rest of the country. Nationwide *Dermatophagoides* sensitization duplicated the sensitization prevalence of the second most frequent allergens: the storage mite *Blomia tropicalis*, cat, cockroach and pollen of Bermuda grass, ash (*Fraxinus excelsior*) and oak (*Quercus ilex*). In the tropical zone HDM allergy was present in approximately 90% of the subjects, consistent with previously published retrospective findings [14].

Many studies have reported sensitization prevalence using skin prick testing. However, the large epidemiologic studies have often encountered problems in standardizing the SPT methodology, resulting in unexplainable differences found in centers within the same region. Moreover, in both large epidemiological SPT studies, the US National Health and Nutrition Examination Survey (NHANES) II and III [18] and the European Community Respiratory Health Survey I [19] investigators’ bias had not been excluded as the skin testing panel was not blinded. With our study design we overcame these pitfalls applying skin prick testing with a blinded panel of allergens, using the same batch, the same device and a standardized method in the centers. The SPT technique was submitted to quality control, as all personnel involved passed a proficiency SPT test before study-start [15]. Also, different from NHANES III and ECRHS-I, our study population was a selected one: patients visiting allergy centers with rhinitis symptoms and a positive routine SPT. Finally, apart from skin testing, the patients also completed a validated allergic rhinitis questionnaire allowing us to categorize the AR symptom phenotype of each patient according to the seasonal-perennial and the ARIA classifications. Consequently, AR phenotypes could be linked to sensitization profiles.

This is the first epidemiologic study to show that in a (sub)tropical region there is no specific relationship between any of the ARIA symptom phenotypes and allergen sensitization. For the old SAR-PAR classification there was a weak association between SAR and certain pollen sensitizations: SAR subjects have 11.5% more tree and 13.3% more grass pollen SPT-positivity. Although there have been epidemiological studies before [20-24] on AR and allergen sensitization, these studies did not report on the relationship between AR symptom phenotypes and specific sensitizations patterns.

Comparing the sensitization profile of AR patients in a (sub)tropical country to those found in SPT positive subjects in Europe (ECRHQ), the frequency of HDM sensitization falls in the same range, that of grass pollen is lower than in Europe, but tree and ragweed pollen sensitization is higher in our subjects [19]. Moreover, the main allergenic grass pollen in Europe was Timothy and is Bermuda grass pollen in our patients. More recently, skin test sensitization in a market in Belgium showed a similar profile as the ECRHQ, but with lower HDM sensitization in this Northern European country [25].

Of all SPT positive subjects in the US epidemiologic census, NHANES-III, SPT positivity to HDM, perennial rye pollen, short ragweed pollen or *Blatella germanica* were all close to 50%. In our selected AR patient population HDM sensitization was higher, but cockroach, grass and ragweed pollen sensitization much lower than in this US census. However, as the potency of the US extracts used in NHANES-III was higher than the potency of the European extracts we used [26,27] and the criterion for a positive test was less stringent in NHANES-III, sensitization frequencies can only be compared relatively.

Apart from *Dermatophagoides spp.* we also tested the storage mite, *Blomia tropicalis*, reported in some subtropical climates to be of importance [28]. Although 25% of our AR subjects had SPT positivity for *B tropicalis*, 93.7% of them were also sensitized to *Dermatophagoides*, leaving only 6.3% of the HDM sensitized subjects, corresponding to 3.8% of the whole population, with specific *B tropicalis* sensitization. Chinese investigators found similar numbers. Analyzing subjects with allergic rhinitis and/or asthma with SPT to HDM and storage mites (SM) –including Blomia tropicalis- 82% had SPT positivity to HDM and% to SM, but only 1.5% of the patients were sensitized to SM without HDM sensitization, as judged by SPT, and 14% according to specific IgE [29]. In a non-selected population of adults in Canary Islands, Spain, SPT positivity for Blomia was as high as 37.2% among those patients with rhinitis symptoms (95% CI 28.1-47.6%) and even 50% among those with asthma (95% CI 26.8-73.2%) [28].

Our observation of increased pollen sensitization in the adult age-group is in agreement with those reported by Asero et al. with respect to the age of onset of ragweed pollen sensitization, situated in the third decade of life [30].

The sensitization pattern partly agrees with the aerobiology of Mexico [31] that shows high concentrations of ash pollen in winter months (November-February), followed by oak and Bermuda grass pollen end-winter into spring. Ash is a species that grows easily under the climate conditions present in a subtropical country. In Mexico City *Fraxinus* is even more abundant, because it is the main tree used in re-forestation projects. Interestingly, sensitization to cypress pollen is not as high as would be expected from the abundant pollen quantity in the air. Worldwide, there is currently a clear tendency towards an increase in atmospheric pollen, including highly allergenic taxa. Experimental studies in a multinational study across Europe suggest that these trends cannot solely be attributed to rising temperatures, but might also be influenced by the increase of the greenhouse gas CO2 [32], an observation definitely of importance in the three major cities of Mexico where pollution is an issue.

In conclusion, in patients with AR symptoms living in the (sub)tropics, SPT sensitization patterns are different from those found in Europe and US. Sensitization patterns are not clearly linked to any specific AR symptom phenotype, but they do vary according to age (child-adolescent vs. adult) and climate zone. Hence, in our population sensitization to a certain allergen is not linked to either intermittent or persistent AR, or to mild or moderate-severe AR.

### IRB approval

This study was approved by Comité de Ética del *Instituto Jalisciense de investigación Clínica SA de CV* on the 12^th^ of January 2010.

## Abbreviations

AR: Allergic rhinitis; ARIA: Allergic rhinitis and its impact on asthma; ECRHS-I: European Community Respiratory Health Survey I; HDM: House dust mite; INEGI: *Instituto Nacional de Estadística y Geografía* (according to its initials in Spanish); IAR: Intermittent allergic rhinitis; NHANES: National Health and Nutrition Examination Survey; PER: Persistent allergic rhinitis; PAR: Perennial allergic rhinitis; SAR: Seasonal allergic rhinitis; SM: Storage mite; SPT: Skin prick test; US: United States of North America.

## Competing interests

The authors declare that they have no competing interests.

## Authors’ contributions

DLL developed the study-design, coordinated the data recollection and analysis and was in charge of the publication writing, submission and review process. HD was involved with the design of the study, the data analysis, review and approval of publication content. KSH designed the randomization and chaired the statistical analysis. He is author of several figures and he contributed to the review and approval of statistical data. AM discussed the design of the study –statistical part- and coordinated the statistical data analysis. RM has had a definite impact on the study design, gave the global ideas for the data analysis, corrected the publication draft and approved its final content. AAC, MAM, MBB, RCJ, MLCP, MACR, RGA, CYGC, DAGI, RGM, DHC, FJLZ, JALP, JJMC, NMJ, MAMA, AMH, AMM, DNL, LJPN, ERS, NRP and PGRO have participated in the details in design per center, data collection per center, data-correction and review and approval of the Mexican data in the publication. All authors read and approved the final manuscript.

## Supplementary Material

Additional file 1: Figure S1 Skin prick test positivity all allergens nationwide: wheal size. Click here for file

Additional file 2: Table S1 Mean wheal size (cm^2^) of pollen extracts in SPT (+) subjects per subgroup. **Table S2.** Mean wheal size (cm^2^) of non-pollen extracts in SPT (+) subjects per subgroup. Click here for file

## Take home message

Sensitisation of Mexicans with allergic rhinitis (AR) 60% *Dermatophagoides* and related to age and climate, not to ARIA classification.

## References

[B1] Ait-Khaled N Pearce N Anderson HR Ellwood P Montefort S Shah J Global map of the prevalence of symptoms of rhinoconjunctivitis in children: The International Study of Asthma and Allergies in Childhood (ISAAC) Phase Three Allergy 2009 64 1 123 148 19132975 10.1111/j.1398-9995.2008.01884.x

[B2] Bousquet J Anto JM Demoly P Schunemann HJ Togias A Akdis M Auffray C Bachert C Bieber T Bousquet PJ Carlsen KH Casale TB Cruz AA Keil T Lodrup Carlsen KC Maurer M Ohta K Papadopoulos NG Roman Rodriguez M Samolinski B Agache I Andrianarisoa A Ang CS Annesi-Maesano I Ballester F Baena-Cagnani CE Basagana X Bateman ED Bel EH Asthma, WHO Collaborating Center for Rhinitis Severe chronic allergic (and related) diseases: a uniform approach–a MeDALL–GA2LEN–ARIA position paper Int Arch Allergy Immunol 2012 158 3 216 231 22382913 10.1159/000332924

[B3] Bousquet J Lockey R Malling HJ Allergen immunotherapy: therapeutic vaccines for allergic diseases. A WHO position paper J Allergy Clin Immunol 1998 102 4 Pt 1 558 562 9802362 10.1016/s0091-6749(98)70271-4

[B4] Cox L Nelson H Lockey R Calabria C Chacko T Finegold I Nelson M Weber R Bernstein DI Blessing-Moore J Khan DA Lang DM Nicklas RA Oppenheimer J Portnoy JM Randolph C Schuller DE Spector SL Tilles S Wallace D Allergen immunotherapy: a practice parameter third update J Allergy Clin Immunol 2011 127 1 Suppl S1 S55 21122901 10.1016/j.jaci.2010.09.034

[B5] Larenas-Linnemann D Ortega-Martell JA Del Rio-Navarro B Rodriguez-Perez N Arias-Cruz A Estrada A Becerril-Angeles M Pietropaolo-Cienfuegos DR Ambriz-Moreno MJ Baez-Loyola C Cossio-Ochoa E Gonzalez-Diaz SN Hidalgo-Castro EM Huerta-Hernandez RE Macias-Weinmann A Oyoqui-Flores J Stone-Aguilar H Trevino-Salinas MB Zarate-Hernandez Mdel C [Mexican clinical practice guidelines of immunotherapy 2011] Rev Alerg Mex 2011 58 1 3 75 21967873

[B6] INEGI 2011 http://mapserver.inegi.gob.mx/geografia/espanol/datosgeogra/climas/climas.cfm (accessed January, 10th 2013)

[B7] Sole D Mallol J Camelo-Nunes IC Wandalsen GF Prevalence of rhinitis-related symptoms in Latin American children - Results of the International Study of Asthma and Allergies in Childhood (ISAAC) phase three Pediatr Allergy Immunol 2010 21 e127 e136 19788538 10.1111/j.1399-3038.2009.00947.x

[B8] Pearce N Ait-Khaled N Beasley R Mallol J Keil U Mitchell E Robertson C, and the Isaac Phase Three Study Group Worldwide trends in the prevalence of asthma symptoms: phase III of the International Study of Asthma and Allergies in Childhood (ISAAC) Thorax 2007 62 9 758 766 17504817 10.1136/thx.2006.070169 PMC2117323

[B9] Gonzalez-Diaz SN Del Rio-Navarro BE Pietropaolo-Cienfuegos DR Escalante-Dominguez AJ Garcia-Almaraz RG Merida-Palacio V Berber A Factors associated with allergic rhinitis in children and adolescents from northern Mexico: International Study of Asthma and Allergies in Childhood Phase IIIB Allergy Asthma Proc 2010 31 4 e53 e62 20819316 10.2500/aap.2010.31.3346

[B10] Lopez Perez G Morfin Maciel BM Huerta Lopez J Mejia Covarrubias F Lopez Lopez J Aguilar G Rivera Perez JL Lopez Medina L Vargas F [Prevalence of allergic diseases in Mexico City] Rev Alerg Mex 2009 56 3 72 79 19623783

[B11] Larenas-Linnemann D Michels A Dinger H Arias-Cruz A Ambriz Moreno M Bedolla Barajas M Cerino Javier R Cid del Prado ML Cruz Moreno MA Diego Vergara L García Almaráz R García-Cobas CY Garcia Imperial DA Garcia Muñoz R Hernandez Colín D Linares Zapien FJ Luna Pech JA Matta Campos JJ Martinez Jimenez N Medina Avalos MA Medina Hernandez A Monteverde Maldonado A López Lizárraga DN Pizano Nazara LJ Ramirez Sanchez E Ramos López JD Rodriguez-Pérez N Rodriguez Ortiz PG Shah-Hosseini K Mösges R In the (sub)tropics Allergic Rhinitis and Its Impact on Asthma classification of allergic rhinitis is more useful than perennial–seasonal classification Am J Rhinol Allergy 2014 28 232 238 doi:10.2500/ajra.2014.28.4035 24980234 10.2500/ajra.2014.28.4035

[B12] Bauchau V Durham SR Epidemiological characterization of the intermittent and persistent types of allergic rhinitis Allergy 2005 60 3 350 353 15679721 10.1111/j.1398-9995.2005.00751.x

[B13] Bousquet J Khaltaev N Cruz AA Denburg J Fokkens WJ Togias A Zuberbier T Baena-Cagnani CE Canonica GW van Weel C Agache I Ait-Khaled N Bachert C Blaiss MS Bonini S Boulet LP Bousquet PJ Camargos P Carlsen KH Chen Y Custovic A Dahl R Demoly P Douagui H Durham SR van Wijk RG Kalayci O Kaliner MA Kim YY Kowalski ML Allergic Rhinitis and its Impact on Asthma (ARIA) 2008 update (in collaboration with the World Health Organization, GA(2)LEN and AllerGen) Allergy 2008 63 Suppl 86 8 160 18331513 10.1111/j.1398-9995.2007.01620.x

[B14] Larenas-Linnemann DE Fogelbach GA Alatorre AM Cruz AA Colin DD Pech JA Hernandez AM Imperial DA del Prado ML Zapien FJ Huerta RE Martell JA Patterns of skin prick test positivity in allergic patients: usefulness of a nationwide SPT chart review Allergol Immunopathol (Madr) 2011 39 6 330 336 21216084 10.1016/j.aller.2010.09.006

[B15] Oppenheimer J Nelson HS Skin testing Ann Allergy Asthma Immunol 2006 96 2 Suppl 1 S6 S12 16496505 10.1016/s1081-1206(10)60895-2

[B16] Hordle DA Mehta V Tomensen B Wainscott G Development of the skin prick test for allergen assay J Immunol Methods 1984 75 2 369 382 6520404 10.1016/0022-1759(84)90121-2

[B17] Service Ns 2012 http://www.nss.gov.au/nss/home.nsf/pages/Sample+size+calculator (accessed 14th of May 2013)

[B18] Arbes SJ Jr Gergen PJ Elliott L Zeldin DC Prevalences of positive skin test responses to 10 common allergens in the US population: results from the third National Health and Nutrition Examination Survey J Allergy Clin Immunol 2005 116 2 377 383 16083793 10.1016/j.jaci.2005.05.017

[B19] Bousquet PJ Chinn S Janson C Kogevinas M Burney P Jarvis D Geographical variation in the prevalence of positive skin tests to environmental aeroallergens in the European Community Respiratory Health Survey I Allergy 2007 62 3 301 309 17298348 10.1111/j.1398-9995.2006.01293.x

[B20] Ibero M Justicia JL Alvaro M Asensio O Dominguez O Garde J Sancha J Valero A Diagnosis and treatment of allergic rhinitis in children: results of the PETRA study Allergol Immunopathol 2012 40 3 138 143 10.1016/j.aller.2010.12.010 21497009

[B21] Anastassakis K Chatzimichail A Androulakis I Charisoulis S Riga M Eleftheriadou A Danielides V Skin prick test reactivity to common aeroallergens and ARIA classification of allergic rhinitis in patients of Central Greece Eur Arch Oto-rhino-laryngology Offic J Eur Fed Oto-Rhino-Laryngological Soc 2010 267 1 77 85 10.1007/s00405-009-1065-x 19690878

[B22] Asha’ari ZA Yusof S Ismail R Che Hussin CM Clinical features of allergic rhinitis and skin prick test analysis based on the ARIA classification: a preliminary study in Malaysia Ann Acad Med Singapore 2010 39 8 619 624 20838703

[B23] Han Y Zhang H [Epidemiological investigation of allergic rhinitis in the primary school students in grade three of Shihezi city] Lin chuang er bi yan hou tou jing wai ke za zhi = J Clin Otorhinolaryngol Head Neck Surg 2009 23 23 1074 1078 20359108

[B24] Antonicelli L Micucci C Voltolini S Feliziani V Senna GE Di Blasi P Visona G De Marco R Bonifazi F Allergic rhinitis and asthma comorbidity: ARIA classification of rhinitis does not correlate with the prevalence of asthma Clin Exp Allergy 2007 37 6 954 960 17517110 10.1111/j.1365-2222.2007.02729.x

[B25] Blomme K Tomassen P Lapeere H Huvenne W Bonny M Acke F Bachert C Gevaert P Prevalence of allergic sensitization versus allergic rhinitis symptoms in an unselected population Int Arch Allergy Immunol 2013 160 2 200 207 23018768 10.1159/000339853

[B26] Larenas-Linnemann D Cruz AA Gutierrez IR Rodriguez P Shah-Hosseini K Michels A Mosges R European and Mexican vs US diagnostic extracts of Bermuda grass and cat in skin testing Ann Allergy Asthma Immunol 2011 106 5 421 428 21530875 10.1016/j.anai.2010.11.020

[B27] Larenas-Linnemann D Matta JJ Shah-Hosseini K Michels A Mosges R Skin prick test evaluation of Dermatophagoides pteronyssinus diagnostic extracts from Europe, Mexico, and the United States Ann Allergy Asthma Immunol 2010 104 5 420 425 20486333 10.1016/j.anai.2010.03.009

[B28] Julia-Serda G Cabrera-Navarro P Acosta-Fernandez O Martin-Perez P Garcia-Bello MA Anto-Boque J Prevalence of sensitization to Blomia tropicalis among young adults in a temperate climate J Asthma 2012 49 4 349 354 22486531 10.3109/02770903.2012.672611

[B29] Zhang C Li J Lai X Zheng Y Gjesing B Spangfort MD Zhong N House dust mite and storage mite IgE reactivity in allergic patients from Guangzhou China Asian Pac J Allergy Immunol 2012 30 4 294 300 23393909

[B30] Asero R Ragweed allergy in northern Italy: are patterns of sensitization changing? Eur Ann Allergy Clin Immunol 2012 44 4 157 159 23092001

[B31] Centro de Ciencias de la A Red Mexicana de Aerobiología (REMA) 2010 http://www.atmosfera.unam.mx/rema/estaciones_muestreo.html# (accessed January 10th 2013)

[B32] Ziello C Sparks TH Estrella N Belmonte J Bergmann KC Bucher E Brighetti MA Damialis A Detandt M Galan C Gehrig R Grewling L Gutierrez Bustillo AM Hallsdottir M Kockhans-Bieda MC De Linares C Myszkowska D Paldy A Sanchez A Smith M Thibaudon M Travaglini A Uruska A Valencia-Barrera RM Vokou D Wachter R de Weger LA Menzel A Changes to airborne pollen counts across Europe PLoS One 2012 7 4 e34076 22514618 10.1371/journal.pone.0034076 PMC3325983

