# Supplementary figures and images for: Allergen sensitization linked to climate and age, not to intermittent-persistent rhinitis in a cross-sectional cohort study in the (sub)tropics
Source: Clin Transl Allergy. 2014 Jun 4;4:20. doi: 10.1186/2045-7022-4-20 (PMC4073512; doi:10.1186/2045-7022-4-20)

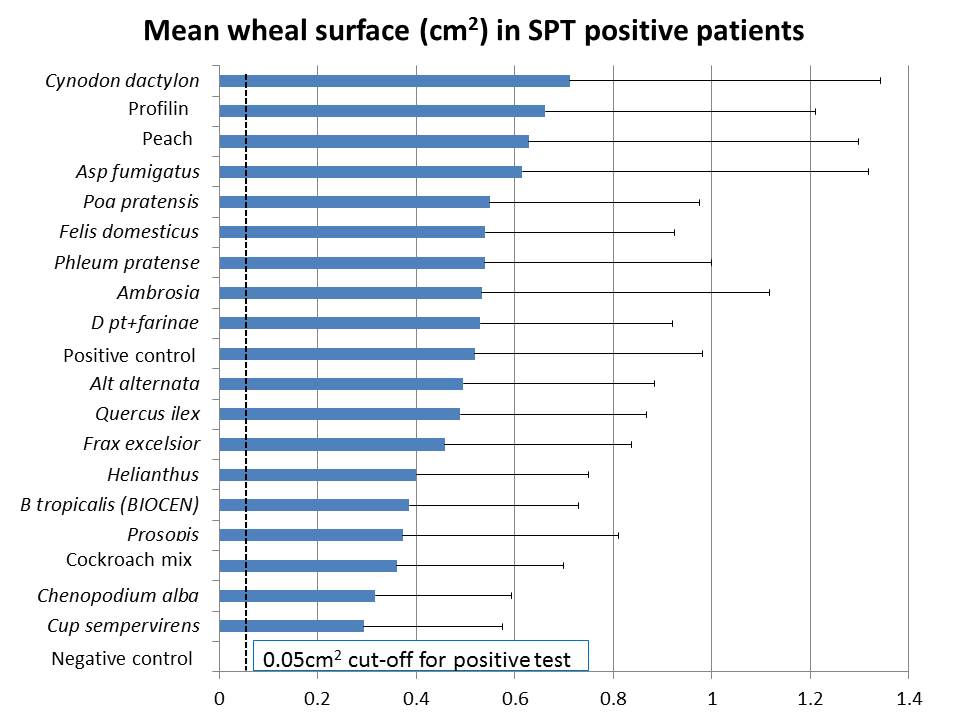

Supplement: Additional file 1: Figure S1 — Skin prick test positivity all allergens nationwide: wheal size. [file 2045-7022-4-20-S1.jpeg]
